# The outcomes of a standardized approach to managing metabolic bone disease of prematurity

**DOI:** 10.1186/1687-9856-2015-S1-O52

**Published:** 2015-04-28

**Authors:** Lit Kim Chin, John Doan, Yvonne Sui Lien Teoh, Alice Stewart, Peter Forrest, Peter J Simm

**Affiliations:** 1 Royal Children’s Hospital, Parkville, Melbourne, VIC, Australia; 2 Department of Paediatrics, Dandenong Hospital, Monash Health, Dandenong, sVIC, Australia; 3 Department of Neonatology, Monash Medical Centre, Melbourne, VIC, Australia; 4 Department of Paediatric Endocrinology and Diabetes, Monash Children’s Hospital, Clayton, VIC, Australia; 5 Murdoch Children’s Research Institute, Parkville, Melbourne, VIC, Australia

## 

Metabolic bone disease (MBD) of prematurity is an increasingly well-recognized complication of pre-term birth. Despite this, there is limited evidence for the optimum method for assessing and monitoring bone health and appropriate supplementation.

This study assesses the effectiveness of the MBD protocol at Monash Health nurseries (Clayton, Dandenong and Casey) in infants born under 32 weeks’ gestation between November 2012 and January 2014.

Preliminary data of 93 infants (mean gestational age (GA) 29 weeks (24-32.3weeks), birth weight (BW) 1279.4g (553-2512g)) were assessed. Risk factors assessed include 8.8% IUGR infants (n=56), 12.9% maternal pre-eclampsia, 7.5% necrotising enterocolitis (NEC) episodes and exposure to medications as follows:caffeine (84.9%; mean 42 days), diuretics (23.7%; mean 13.8 days) and steroids (2.2%). Preterm infants received 14.3 days of total parenteral nutrition (TPN) on average, with the majority on fortified expressed breast milk once enteral feeding was established.

Initial MBD screen was performed for 80.6% infants (mean age 36.6 days) with only 24.7% having repeated monitoring (mean age 67.9 days). 6.5% had Alkaline phophosphatase (ALP) levels >500U/I initially (range 143-827U/l), reducing to 1.1% (range 173-573U/I). Average Tubular Resorption of Phosphate (TRP) was 79.7% (n=71). The majority of infants were on Vitamin D 400units/day. 23.6%(n=22) commenced phosphate supplements (mean duration 41 days) and 15.1%(n=14) commenced calcium supplements (mean duration 53.2 days).

Average birth length was 38cm (10-50^th^centile) with evidence of slowing growth velocity (mean follow up length 49cm (<3^rd^centile) at mean age 79.9 days). Five infants were identified with fractures however two were from birth trauma and two suspected non-accidental injury. One patient had an incidental finding of fractured femur with multiple risk factors for MBD including very low birth weight (700g), NEC episodes requiring prolonged antibiotic therapy (69 days), TPN (48 days), caffeine (88 days) and diuretic use (22 days).

Significant difference (p<0.001) is noted between phosphate-treatment and untreated groups for both GA and BW: Median 27weeks and 929g for treated subjects versus 29.6weeks and 1343g if untreated. In the phosphate-treatment group, ALP levels improved (mean pre-treatment414U/l and post-treatment264U/l, p=0.0006) and difference in phosphate levels were also significant with p=0.003. Between phosphate-treatment group versus untreated group, differences were insignificant for ALP (p=0.05) and phosphate levels (p=0.09), though this may reflect insufficient subsequent MBD screens (treatment group, n=15 versus untreated group, n=7).

Further evaluation is anticipated to help improve MBD understanding in this high-risk cohort particularly given morbidity associated with MBD occult fractures. In addition, secondary outcomes would include cost efficiency of MBD surveillance and identifying optimal supplemental therapy.

